# Supplementary material for: Rapid turnover of life-cycle-related genes in the brown algae
Source: Genome Biol. 2019 Feb 14;20:35. doi: 10.1186/s13059-019-1630-6 (PMC6374913; doi:10.1186/s13059-019-1630-6)
Supplement: Supplementary file 3 — Protein alignments, corresponding Gblocks html file and CDS sequences for the lineage conserved gametophyte-biased genes. (ZIP 2109 kb) [file 13059_2019_1630_MOESM3_ESM.zip › Con_GA_alignments/Ec-02_005700.aa_ali.fasta-gb.htm]

Ec-02\_005700.aa.fasta.trans.aa\_ali.fasta


## Gblocks 0.91b Results

Processed file: **Ec-02\_005700.aa.fasta.trans.aa\_ali.fasta**  
Number of sequences: **2**  
Alignment assumed to be: **Protein**  
New number of positions: **320** (selected positions are underlined in blue)

```
                         10        20        30        40        50        60
                 =========+=========+=========+=========+=========+=========+
Ec-02_005700     MVLLLAWTTYFGAGTLRRCKDWRSERTLFESAFKVCPDGIKTLNNLAVGMLNEKEAGRAE
MSTRG.23926.1    ------------------------------------------------------EAGRAQ
                                                                       ######


                         70        80        90       100       110       120
                 =========+=========+=========+=========+=========+=========+
Ec-02_005700     ELLRRAVELHPDFGFAQFNLGVSLMIQRDHLGAVSALERSLRLEPSNTKVMVYLGQVYLE
MSTRG.23926.1    VLLRRAVELHPHFGFAFFNLGVSLKITRDHVGAVSAFERSLEVEPWNTKVMVLLGEVYLE
                 ############################################################


                        130       140       150       160       170       180
                 =========+=========+=========+=========+=========+=========+
Ec-02_005700     LTLKRLEPLPQRDH-GNGVDAGFDDTQHHDG-GAIAGDGQTDASNDMRSSSAESLLSSAE
MSTRG.23926.1    LALTALETSTAGAHRGTEVNSDSDDARHQSVVDGIYDDGGHAAR--IRSSPPESLLASAE
                 ############## #############                   #############


                        190       200       210       220       230       240
                 =========+=========+=========+=========+=========+=========+
Ec-02_005700     LNTDRALAAGTGLPLARLVKGQALALRGQHQSAAKFFQESIDISRRLLSGRS-----AAK
MSTRG.23926.1    FYTDRALDAGSGLPLARLVKGHVLSLRGQHQLAAAFFQESIDLSLHLLSRRSGTIAFKAA
                 ####################################################      ##


                        250       260       270       280       290       300
                 =========+=========+=========+=========+=========+=========+
Ec-02_005700     VRNNESNNPVIAAETIEVADAVDLASTYNRLGLTLRDSGEDLVATKEAFLAGLSVTPDDL
MSTRG.23926.1    SSKDDSISVWAAAGSMEASDAIDVASTYNRLGLALRDSGGDVDAAKEAFLAGLSVAPDNL
                 ############################################################


                        310       320       330       340       350       360
                 =========+=========+=========+=========+=========+=========+
Ec-02_005700     ALLVNGGVAHQTAGDIPGAKALFHRALSLQPNSPELLNNLGWLEEQTGGGSKLSLETAAG
MSTRG.23926.1    ALLVNGGVVHQNAGDIPGAKALYLRALSLQPNSPELLSNMGWLEEQSGGGSRLSLETAAE
                 ############################################################


                        370       380       390       400       410
                 =========+=========+=========+=========+=========+=========
Ec-02_005700     LYERAIRLLDERSPVLGRVEANLSSVQNRLLLKEVEDWPPATAKIDERLEDPYYFVNRQ
MSTRG.23926.1    MYDRALVLLDEESPALEQVETNARNVRNRLLLMNDGQLSP------EGL---------E
                 ########################################
```

```
Parameters used
Minimum Number Of Sequences For A Conserved Position: 2
Minimum Number Of Sequences For A Flanking Position: 2
Maximum Number Of Contiguous Nonconserved Positions: 8
Minimum Length Of A Block: 10
Allowed Gap Positions: None
Use Similarity Matrices: Yes
```

```
Flank positions of the 4 selected block(s)
Flanks: [55  134]  [136  148]  [168  232]  [239  400]  

New number of positions in Ec-02_005700.aa.fasta.trans.aa_ali.fasta-gb:  320  (76% of the original 419 positions)
```
